# A QM-AI Approach for the Acceleration of Accurate Assessments of Halogen‑π Interactions by Training Neural Networks

**DOI:** 10.1021/acs.jcim.5c02136

**Published:** 2025-11-25

**Authors:** Marc U. Engelhardt, Finn Mier, Markus O. Zimmermann, Frank M. Boeckler

**Affiliations:** † Laboratory for Molecular Design & Pharmaceutical Biophysics, Institute of Pharmaceutical Sciences, Department of Pharmacy and Biochemistry, 9188 Eberhard Karls Universität Tübingen, 72076 Tübingen, Germany; ‡ Interfaculty Institute for Biomedical Informatics (IBMI), Eberhard Karls Universität Tübingen, 72076 Tübingen, Germany

## Abstract

Noncovalent interactions, such as halogen bonds (XB), play a crucial role in molecular recognition and drug design, yet halogen···π contacts remain comparatively underexplored. Here, we report a proof-of-concept QM-AI approach that integrates high-level quantum mechanical (QM) calculations with neural networks (NNs) to predict halogen···π interaction energies. Nearly 1.4 million MP2/TZVPP single-point calculations on halobenzene–benzene complexes were carried out to generate exhaustive training data, which were represented by simple geometric descriptors as input features for machine learning. The resulting neural network model is specifically designed to capture σ-hole-driven halogen···π interactions under well-defined geometric constraints. The resulting model reproduced reference interaction energies with excellent accuracy (*R* ^2^ = 0.998, RMSE = 0.16 kJ/mol) and maintained strong performance on independent, randomly generated and PDB-derived test sets. Previously, we have demonstrated in a benchmarking study that “gold standard” CCSD­(T) energies of this interaction can be appropriately represented by MP2/TZVPP calculations, but at a better calculation efficiency by 2 orders of magnitude (∼10^2^). Consequently, we herein exploit a methodological “extension” from CCSD­(T) → MP2 → NNs. Our approach maintains accuracy close to CCSD­(T) benchmarks while achieving a runtime acceleration of up to 8 orders of magnitude (∼10^8^) compared to MP2 calculations. This study demonstrates the feasibility of fast, accurate neural network models based on QM data for halogen···π interactions in a QM-AI approach.

## Graphical abstract

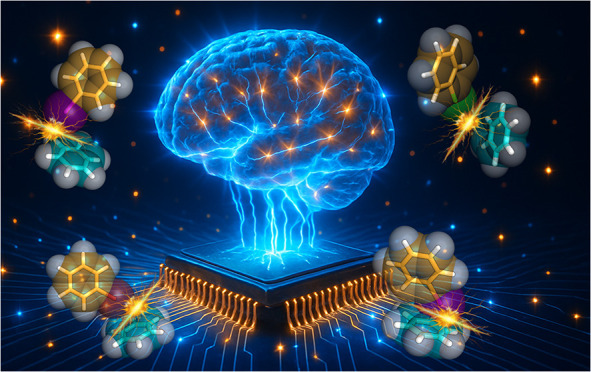

## Introduction

Noncovalent interactions are central to a wide range of processes in biological systems and molecular recognition, such as protein folding, drug binding, and protein–ligand interactions. In particular, understanding the individual contributions is crucial for elucidating biomolecular functions and guiding the rational design of potential therapeutics.^1–5^ Among diverse types of noncovalent interactions, halogen bonding (XB) has emerged as a powerful and versatile tool due to its high directionality and ability to interact with a multitude of different partners in the protein binding site. It is defined by a directional attraction between a nucleophilic site and an electrophilic region, the σ-hole, typically located on the elongation of the R–X axis on a halogen atom such as chlorine, bromine, or iodine.^6–11^ The highly anisotropic electron distribution around halogen atoms results in a significant lateral electron density, oriented perpendicular to the R–X bond axis. This feature contributes to the characteristically pronounced directionality of halogen bond interactions. In systems where the substituent R exerts a strong electron-withdrawing effect on the halogen (X), such “tuning effects” can significantly enhance the strength of the halogen bond.^12–15^ Accordingly, the strategic incorporation of halogen bonds in drug design has attracted increasing attention to optimize pharmacological profiles by enhancing ligand binding affinity or improving specificity and stability in protein–ligand complexes.^16–33^

Computational methods have been extensively applied to accurately characterize the geometric and energetic features of halogen bonding. To date, halogen bonds have been systematically investigated in the context of various nucleophilic moieties within binding sites, including backbone carbonyl groups and the π-surface of the peptide bond,^34,35^ the sulfur atom in methionine,^36^ the nitrogen atoms in histidine,^37^ carboxylate groups of aspartate and glutamate, as well as carboxamide groups of asparagine and glutamine moieties,^38^ and the oxygen atoms of water molecules.^39,40^ In contrast, halogen bonding involving the electron-rich π-system of the aromatic side chain residues of phenylalanine, tyrosine, histidine, and tryptophan, however, remains comparatively unexplored in the context of protein–ligand interactions.^41–45^ In a very recent study, we used quantum mechanical calculations to examine halogen···π interactions between halobenzenes (strongly focusing on iodine interactions) as ligand systems and benzene as a surrogate for the aromatic side chain of phenylalanine.^46^ We compared calculations of different QM methods and basis sets and showed that calculations on an MP2 level of theory using a reasonably large basis set of TZVPP or cc-pVTZ yield highly accurate energies compared to reference calculations (CCSD­(T)/CBS_extrapolation_) while maintaining feasible computational demands, even for larger data sets. This transition from CCSD­(T) to MP2 corresponds to a computational speed-up of about 2 orders of magnitude (∼10^2^) without a significant loss in accuracy. This enabled us to generate the basis for the following machine learning approach, aiming for a tremendous speed-up to an almost instant geometric assessment of high quality energies of halogen···π interactions.

The computational complexity of QM methods can become a significant obstacle, when applied to larger systems or when evaluating large data sets. To address this limitation, data-driven approaches such as neural networks (NNs) are emerging as powerful tools to complement or even replace QM calculations in certain contexts.^47–50^ One of the key strengths of NN-based approaches is their scalability and versatility. Once trained, models can predict interaction energies for a wide range of molecular systems in a tiny fraction of the time required for QM calculations. Inputs to these NNs are typically molecular descriptors representing crucial geometric features such as interaction distances and angles. Thus, the integration of QM-calculated data sets with NN-based models represents a promising approach to bridge the gap between computational cost and accuracy.^51–53^

Shaw et al.^54^ introduced a simple two-parameter statistical model to predict halogen bond interaction energies for a small data set of halogenated compounds. Using this basic type of machine learning model on an unseen test set of 80 complexes, they achieve accuracies within ∼2.1 kJ/mol emphasizing the applicability of machine learning in general. In 2023, Samuel et al.^55^ published a general overview of various machine learning approaches on halogen bonding and their differences concluding that it is a “...powerful tool for unravelling the intricacies of molecular interactions and guiding the design of functional molecular systems”. They foresee continued progress as larger data sets become more available, and hybrid quantum-machine learning (“QM-AI”) approaches become more prevalent. Devore et al.^56^ published a machine learning-based approach to characterize halogen bonding interactions using molecular fingerprints as input descriptors. Their study showed that supervised learning models can accurately classify halogen-bond donor types and predict interaction properties, further highlighting the potential of data-driven techniques to complement QM methods in evaluating halogen bonding complexes.

In this study, we focus on the systematic investigation of halogen···π interactions using high-level quantum mechanical (QM) methods and integrating them into NN models. Based on findings of our previous study, a data set of interaction geometries composed of halobenzenes (chlorobenzene, bromobenzene, and iodobenzene) in complex with benzene is generated and used in single point calculations on an MP2/TZVPP level of theory. To specifically investigate and assess σ-hole interactions, a systematic grid of interaction geometries was generated in planes parallel to the π-plane. Each plane was positioned at a fixed halogen···π-plane separation, with distances ranging from *d*_min(X···π‑plane)_ = 2.75 Å to *d*_max(X···π‑plane)_ = 4.50 Å. In this distance definition (*d*_X···π‑plane_) the respective point on the π-plane is individually determined by the normal onto the π-plane through the halogen atom. Within each plane, geometries were sampled to include orientations deviating by no more than 40° between the C–X bond vector and the normal onto the benzene molecule (π-plane). This setup ensures that the data set selectively represents configurations characteristic of σ-hole interactions, while reducing contributions of secondary interactions, e.g π···π, or C–H···π interactions. The results are then used to train NN models in a supervised learning approach to predict adduct formation energies with high accuracy and significantly reduced computational cost. We demonstrated that MP2 reproduces CCSD­(T) reference data with excellent accuracy. This close agreement indicates that MP2 captures the essential properties of these noncovalent interactions, while offering a substantial computational speed-up of approximately 2 orders of magnitude (10^2^) compared to CCSD­(T). Building on this finding, we propose a methodological “double jump” approach, first from CCSD­(T) to MP2, and then from MP2 to machine-learning (NN) models. In the first “jump”, CCSD­(T) → MP2, we replace the computationally prohibitive CCSD­(T) calculations with MP2, achieving near-CCSD­(T) accuracy at a fraction of the cost. In the second “jump”, MP2 → NNs, we train neural network models on extensive MP2 data sets, enabling the prediction of interaction energies with a further acceleration of approximately 8 orders of magnitude (10^8^) compared to the underlying MP2 computations. Together, this “double jump” preserves the accuracy hierarchy established between CCSD(T) and MP2, while extending it further into the field of machine learning. As a result, the NN models inherit the near-CCSD­(T) accuracy of MP2 but with unprecedented computational efficiency. A PDB^57^ scan provided examples from real-world biological systems to evaluate the model and its potential to quickly predict halogen bond interaction energies. By integrating QM calculations with machine learning techniques, this work serves as an initial step to set the stage for the generation of subsequent QM-AI hybrid models able to assess interactions between halogens and other acceptor systems. Ultimately, we aim to employ these models into the molecular docking framework PLANTS, to enhance the identification and scoring of halogen bond interactions in protein–ligand complexes.

## Results and Discussion

### NN Model Training and Validation

First, we conducted almost 1.4 million single-point calculations of the generated interaction geometries. Energies were calculated using the supermolecular approach. Features were derived from the individual interaction geometries. A complete list of all features with their descriptions, along with a more detailed schematic of the feature definitions, is provided in the Supporting Information (Figure S1 and Table S1). To ensure the feature space is translationally and rotationally independent from the underlying coordinate system, we chose pairwise distances and angles. The resulting data set was partitioned into training, validation, and test subsets (Figure 8). Final model training was conducted using the training subset, with performance evaluation on the validation subset at the end of each epoch. Figure 1a illustrates the progression of model performance on the training and validation data set over consecutive epochs. Training was terminated after 276 epochs upon satisfaction of the early stopping criterion. The results of the final evaluation step on the validation set are presented in Figure 1b, where the predicted adduct formation energies are plotted against the calculated values. The model achieved a coefficient of determination *R* ^2^ = 0.9979 and a root-mean-square error RMSE_val_ = 0.1579 kJ/mol, indicating excellent predictive accuracy. Results on the test set also show excellent accuracies with minimal amounts of large energy differences ΔΔ*E* (Figure 2a). Results are given for each halogen individually. Most differences lie within ±0.5 kJ/mol with only a minimal fraction of 1.8% in total lying beyond. An overall maximum of ΔΔ*E* = +2.40 kJ/mol and a minimum of ΔΔ*E* = −2.35 kJ/mol was observed. Overall, the model achieved an *R* ^2^ = 0.9979 and an RMSE_test_ = 0.1590 kJ/mol on the test set, indicating that the predictive accuracy is maintained even on previously unseen data (Figure 2b). The observed performance may be inflated due to similarities between the training and test sets, potentially introducing data leakage as the data points likely reside within the same feature space. Consequently, a more profound evaluation involves assessing the model’s generalization capability on entirely unknown data that fall outside the distribution of the training set.

**Figure 1:**
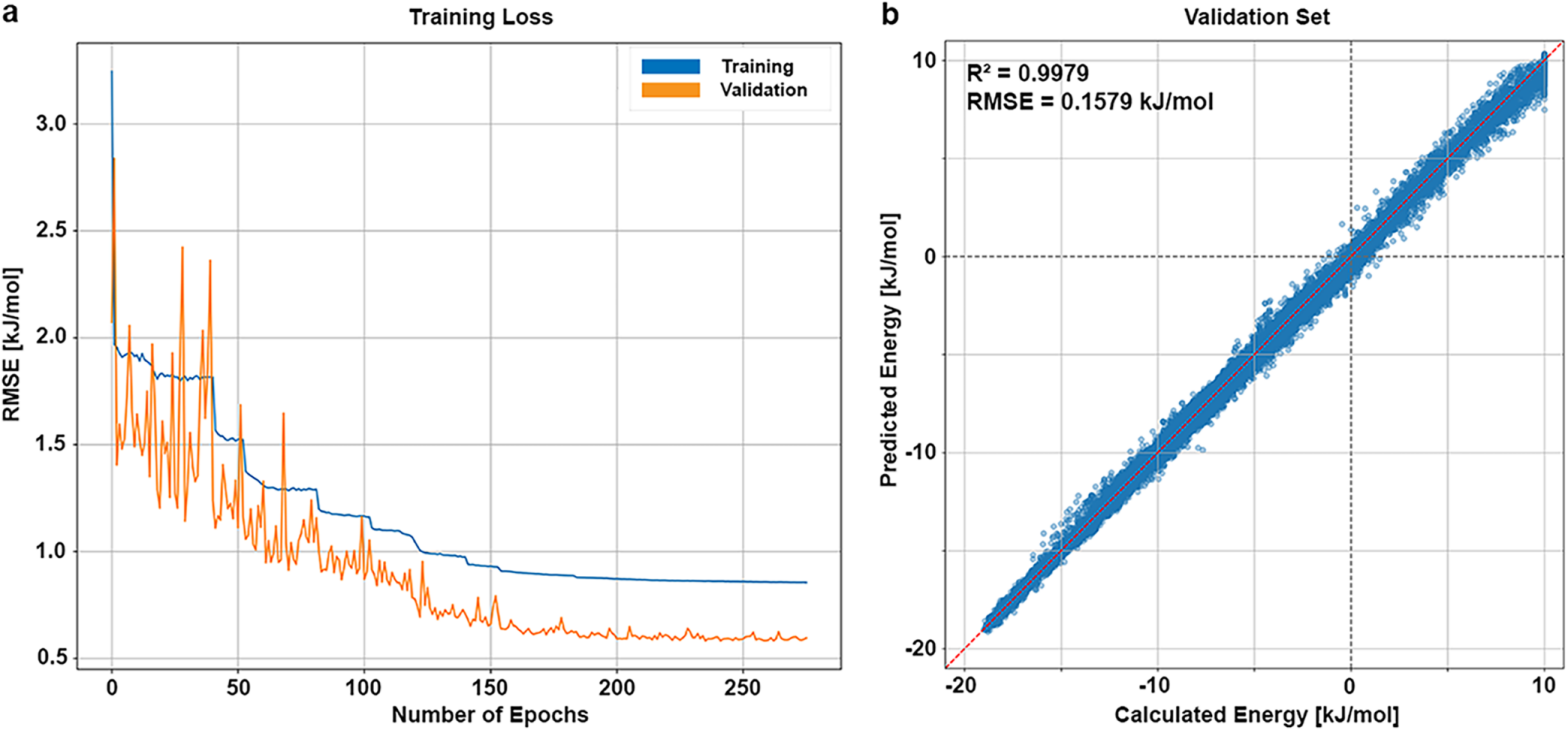
Results of the training process of the final model. (a) RMSE values in kJ/mol for each training epoch. The blue values represent the RMSE for each epoch of the training process, while orange values represent the RMSE of the validation after each training epoch. (b) Final model performance on the validation set with a coefficient of determination of *R*_val_^2^ = 0.9979, and root-mean-square error of RMSE_val_ = 0.1579 kJ/mol. The calculated energy is plotted against the predicted energy. The red, dashed line indicates the perfect correlation between calculated and predicted value, while the gray dashed lines indicate the transition from negative to positive energies.

**Figure 2:**
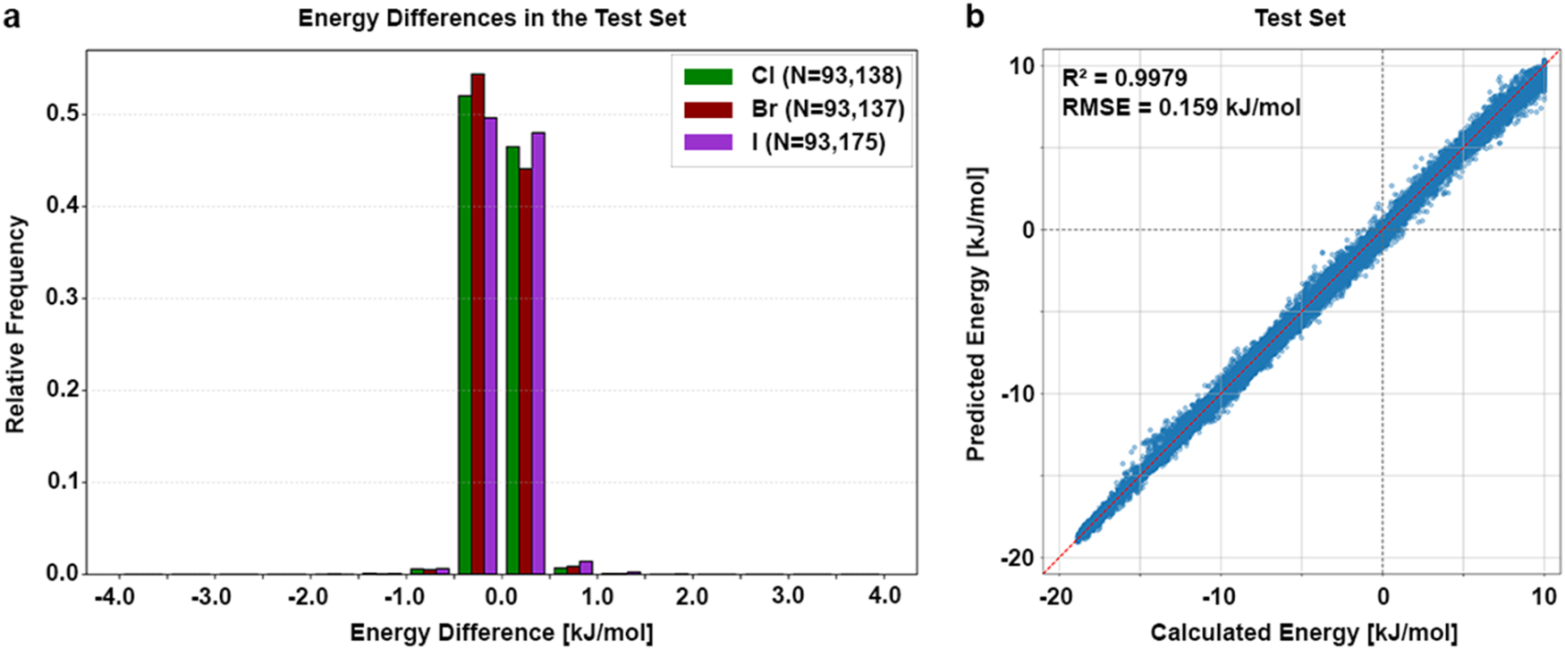
Model performance on the test set. (a) The histogram shows the relative frequencies of energy differences between calculated and predicted energy (total of 279,450 data points) in bins of 0.5 kJ/mol from −4.0 to 4.0 kJ/mol for chlorine (green, *N* = 93,138), bromine (dark red, *N* = 93,137), and iodine (purple, *N* = 93,175) separately. Energy differences are calculated as ΔΔE = Δ*E*_calc_ – Δ*E*_pred_. Larger values are clipped to the respective limitation for better visibility. (b) Calculated energy is plotted against the predicted energy. The model achieved an *R* ^2^ = 0.9979 with an RMSE = 0.159 kJ/mol. The red, dashed line indicates the perfect correlation between calculated and predicted value, while the gray dashed lines indicate the transition from negative to positive energies.

The geometric inclusion criteria employed in the subsequently used data sets of randomly generated and PDB-derived geometries (next sections) were defined with relatively broad angular and distance limits. This approach was chosen to capture a diverse range of halogen···π interaction geometries, including those near the characteristic boundaries of σ-hole interactions. While more restrictive definitions would exclude such borderline geometries and reduce statistical outliers, they would also narrow the physical diversity of the data set and potentially obscure the model’s limitations. Our aim is to evaluate the model’s generalizability across a wide spectrum of interaction geometries, rather than to optimize its performance under tightly constrained conditions. This broader definition also preserves the size and representativeness of the experimentally derived PDB data set, which would otherwise have been substantially reduced by stricter geometric filters applied to the already small data set.

### Prediction of Adduct Formation Energies on Unseen Data

A data set of interaction geometries with randomly chosen translational and rotational features was generated. For a total data set of 30,000 geometries, single point calculations on the MP2/TZVPP level of theory were conducted. Positive energies of more than +10 kJ/mol (repulsions) were excluded, resulting in a final set of 26,481 complexes. Geometric features were extracted from each complex and fed into the model. A detailed table incorporating the individual data points, energies *E*_calc_ and *E*_pred_, as well as the difference between both is provided in spreadsheet format in the Supporting Information. The distribution of energy differences between the calculated and predicted energies is illustrated in Figure 3a. Results are shown for each halogen individually. Most differences still lie within ±0.5 kJ/mol. Approximately 14% of the data points lie beyond ±0.5 kJ/mol, with a maximum of ΔΔ*E* = +27.02 kJ/mol and a minimum ΔΔ*E* = −9.62 kJ/mol. Such outliers are of high interest, because the question arises where they originate from and why the model’s prediction on these data points is poor. Different possibilities can be considered when analyzing outliers in a model’s predictions: (i) The features of the outlier lie outside the range covered by the training data. In this case, poor predictive performance may indicate that the model lacks generalizability and is unable to extrapolate to previously unseen feature spaces. (ii) The features of the outlier lie within the distribution of the training data, but the model still fails to predict accurately. This suggests a deficiency in the model itself, possibly due to overfitting, underfitting, or an insufficiently expressive model architecture. This raises concerns about the model’s internal representation and robustness. To further investigate these outliers and examine their deviation from the expected model behavior, a scatter plot of the calculated and the predicted energy values was generated (Figure 3b). The coefficient of determination *R* ^2^ = 0.856 and the root-mean-square error RMSE = 1.33 kJ/mol confirm the model’s overall strong accuracy. The plot highlights individual deviations across the test set with a color scale indicating the relative Mahalanobis distance (MD), an estimate of how far a given data point lies from the center of the feature distribution. The MD accounts for correlations between input features and scales the distance based on the covariance structure of the data, making it well-suited to identify outliers in multivariate spaces. In this context, a higher MD suggests that the corresponding data point differs substantially from the typical feature profiles seen during training. The majority of the data points lie close to the diagonal, indicating good agreement between predicted and calculated values and consistent with the narrow energy differences observed in Figure 3a. Notably, data points that deviate more strongly from the parity line also tend to exhibit higher MD, suggesting a reasonable correlation between poor predictive performance and a greater dissimilarity from the training data distribution. Additionally, several data points (labeled A–F) show significant deviation and are marked as prominent outliers for illustrative purposes. These points serve as examples for the error sources discussed above and are looked at in detail. Calculated and predicted adduct formation energies, energy differences, distances *d*_X···π‑plane_ between the halogen and the π-plane, as well as the angle between the C–X vector and the normal to the π-plane α_C–X···⊥(π‑plane)_ of these examples are listed in Table 1. Figure 4 visualizes the corresponding interaction geometries. The halogen···π-interactions in Figure 4A–C all feature geometries, where the halogen lies near the edge of the model’s training grid dimensions (illustrated as the teal-colored plane through the benzene), with a C–X vector pointing away from the π-plane, while engaging in potential π···π interactions. The geometry of example A shows an iodobenzene molecule in complex with benzene and is characterized by a short distance of *d*_I···π‑plane_ = 1.70 Å and an angle of α_C–I···⊥(π‑plane)_ = 59.2°. A large energy difference ΔΔ*E*(A) = −9.11 kJ/mol (Δ*E*_calc_ = −23.77 kJ/mol, Δ*E*_pred_ = −14.66 kJ/mol) indicates that the model significantly underestimates the interaction energy. Geometric features likely fall outside the typical range of the feature space covered by the training, as indicated by an MD more than three times higher than that of the feature distribution center. A direct comparison with the training data supports this assumption. The distance in example A *d*_I···π‑plane_(A) = 1.70 Å is substantially shorter than the minimum value in the training set *d*_min(X···π‑plane)_(training) = 2.75 Å, and the angle α_C–I···⊥(π‑plane)_(A) = 59.2° exceeds the maximum training value of α_max(C–X···⊥(π‑plane))_(training) = 40°. Furthermore, the halogen’s proximity to the benzene plane, and thus its in-plane hydrogen atoms, may also contribute beneficially due to contacts between the negatively charged belt of the halogen atom and nearby hydrogen atoms. Similar observations can be found in B and C with underestimations of the interaction energy for both examples (ΔΔ*E*(B) = −8.59 kJ/mol, ΔΔ*E*(C) = −8.6 kJ/mol). Distance and angle values also show significant deviations from the training features with similar geometric attributes. Such errors suggest that the model lacks prior information about π···π interaction motifs or X···H motifs with such compact and tilted geometries, leading to a systematic underestimation of their energetically favorable contribution. Looking at examples D–F, the spatial arrangements change from π···π to C–H···π contributions. With positive energy differences of ΔΔ*E*(D) = 13.43 kJ/mol, ΔΔ*E*(E) = 24.45 kJ/mol, and ΔΔ*E*(F) = 26.62 kJ/mol, the model significantly overestimates these interactions. Halogen atom positions lie close to the edges of the grid dimensions while the halobenzene is oriented above the benzene. While the distance of example D, *d*_I···π‑plane_(D) = 2.73 Å, shows direct proximity to the minimum training distance, the angle value α_C–I···⊥(π‑plane)_(D)= 59.7° still deviates significantly from the training set. However, the acceptable distance, combined with a reasonable C–H···π contact yields a calculated energy of Δ*E*_calc_(D)= −5.5 kJ/mol, while the model predicts Δ*E*_pred_(D)= −18.93 kJ/mol. Example E and F show decreased distances of *d*_I···π‑plane_(E) = 2.45 Å, and *d*_I···π‑plane_(F) = 1.76 Å, respectively. Still observing high angle values, the decreased distance leads to repulsive interactions, with Δ*E*_calc_(E) = 6.24 kJ/mol, and Δ*E*_calc_(F) = 9.89 kJ/mol. In summary, all six outlier examples (A–F) lie outside the range of features represented in the training set, as reflected by their high MDs. Examples A–C exhibit geometries characteristic of close π···π interactions, which are not well represented in the training data. As a result, the model fails to properly capture these types of interaction and underestimates their energetic contributions. In contrast, examples D–F involve very close C–H···π contacts leading to repulsions that are not precisely accounted for in the model. Since such geometries were not explicitly included in the training set, the model naturally lacks the necessary information to recognize and appropriately penalize these interaction patterns. However, the model’s performance on repulsive geometries is not crucial, as such interactions are typically identified and filtered out during earlier stages of the scoring process or by separate repulsive terms. Still, incorporating representative repulsive configurations of σ-hole interactions in future training could further improve the model’s completeness and transferability. The same applies to recognition of π···π interactions. The model will be explicitly used for scoring halogen···π interactions with respect to the σ-hole interacting with aromatic moieties.

**Figure 3:**
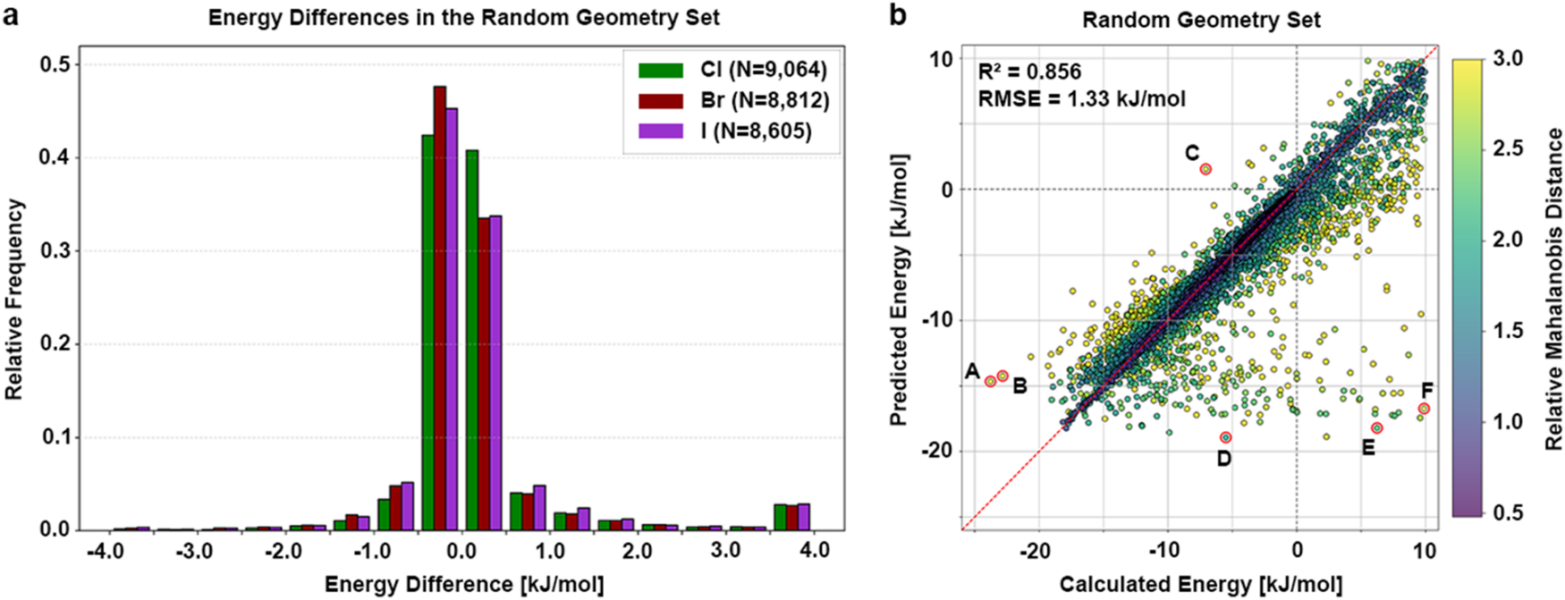
Model performance on the random geometry set. (a) The histogram shows the relative frequencies of energy differences between calculated and predicted energy (total of 26,481 data points) in bins of 0.5 kJ/mol from −4.0 to 4.0 kJ/mol for chlorine (green, *N* = 9064), bromine (dark red, *N* = 8812), and iodine (purple, *N* = 8605) separately. Energy differences are calculated as ΔΔ*E* = Δ*E*_calc_ – Δ*E*_pred_. Larger values are clipped to the respective limitation for better visibility. (b) Calculated energy is plotted against the predicted energy. The model achieved an *R* ^2^ = 0.856 with an RMSE = 1.33 kJ/mol. The red, dashed line indicates the perfect correlation between calculated and predicted value, while the gray dashed lines indicate the transition from negative to positive energies. Each data point is colored according to its relative MD with respect to the given color scale. Data points outlined with a red circle and labeled with (A–F) are shown in detail in Figure 4.

**Figure 4:**
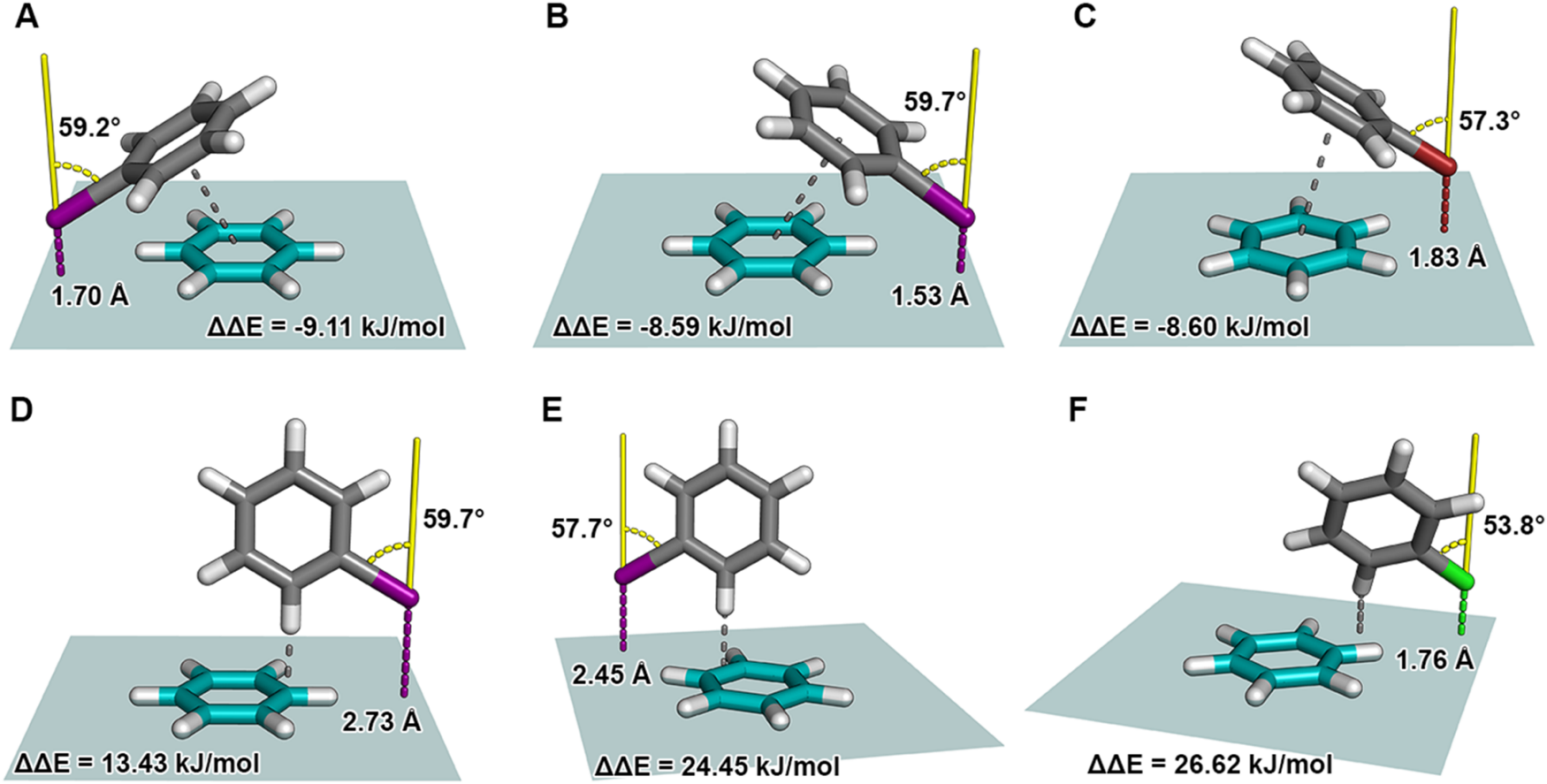
Interaction geometries corresponding to selected outliers of the random geometry set identified in the scatter plot shown in Figure 3. These structures represent data points with high deviations between calculated and predicted adduct formation energies. Each geometry (A–F) illustrates the spatial arrangement of the halobenzene (gray) relative to the π-system of benzene (teal), including the halogen distance *d*_X···π‑plane_ (dashed line, colored according to the halogen) to the π-system plane in Å, the torsion angle α_C–X···⊥(π‑plane)_ (colored yellow) in degrees between the C–X vector and the normal of the π-system plane, as well as the energy difference ΔΔ*E*. The gray dashed line highlights the type of interaction that may contribute to the observed prediction error. For better visibility, the teal-colored plane illustrates the benzene plane and the dimensions of the model’s training grid. (A) Iodobenzene interaction with *d*_I···π‑plane_ = 1.70 Å and α_C–I···⊥(π‑plane)_ = 59.2°. The gray dashed line indicates a π···π-interaction. (B) Iodobenzene interaction with *d*_I···π‑plane_ = 1.53 Å and α_C–I···⊥(π‑plane)_ = 59.7°. The gray dashed line indicates a π···π-interaction. (C) Bromobenzene interaction with *d*_Br···π‑plane_ = 1.83 Å and α_C–Br···⊥(π‑plane)_ = 57.3°. The gray dashed line indicates a π···π interaction. (D) Iodobenzene interaction with *d*_I···π‑plane_ = 2.73 Å and α_C–I···⊥(π‑plane)_ = 59.7° The gray dashed line indicates a C–H···π contact. (E) Iodobenzene interaction with *d*_I···π‑plane_ = 2.45 Å and α_C–I···⊥(π‑plane)_ = 57.7°. The gray dashed line indicates a C–H···π contact. (F) Chlorobenzene interaction with *d*_Cl···π‑plane_ = 1.76 Å and α_C–Cl···⊥(π‑plane)_ = 53.8° The gray dashed line indicates a C–H···π contact.

**Table 1:** Overview of Energy Values and Geometric Parameters for Outlier Structures (A–F) (from the Random Geometry Set) Highlighted in Figure 3 and Depicted in Figure 4 ^a^

	halogen	Δ*E*_calc_ [kJ/mol]	Δ*E*_pred_ [kJ/mol]	ΔΔ*E* [kJ/mol]	*d*_X···π‑plane_ [Å]	α_C–X···⊥(π‑plane)_ [deg]
A	I	–23.77	–14.66	–9.11	1.70	59.18
B	I	–22.83	–14.24	–8.59	1.53	59.66
C	Br	–7.06	1.54	–8.60	1.83	57.28
D	I	–5.50	–18.93	13.43	2.73	59.69
E	I	6.24	–18.21	24.45	2.45	57.69
F	Cl	9.89	–16.73	26.62	1.76	53.82

a The halogen symbolizes the interacting halobenzene. Values of calculated and predicted energies, as well as the difference between both are given in kJ/mol. Distance values *d*_X···π‑plane_ between the halogen and the π-plane are given in Å. Angle values between the C–X vector and the normal to the π-plane are given in degree [deg].

### PDB Scan for Halogen···π Interactions in Crystal Structures

To apply the model to real-world biological examples, a PDB scan was conducted where we focused on protein–ligand recognition from a drug discovery perspective, thus excluding all types of halogenated biomolecular building blocks. 239,149 crystal structures (as of July 2025) were analyzed with 9810 (4.1%) unique structures containing ligands that bear chlorine, bromine, or iodine connected to an aromatic moiety. Initially, halogen···π contacts were considered if the aromatic side chain residue of phenylalanine, tyrosine, histidine, or tryptophan were located within 5 Å of the halogen atom. Results of the PDB scan are summarized in Table 2. A total of 23,536 contacts in 6796 unique PDB structures were found.

**Table 2:** PDB Scan Results for Halogen···π Contacts and Halogen···π Interactions^a^

addressed AA	halogen···π contacts within 5 Å (% of total)	halogen···π interaction after applied filters (% of contacts)
histidine	3597 (15.28%)	661 (18.38%)
**phenylalanine**	**10,174 (43.23%)**	**1114 (10.95%)**
tryptophan	2855 (12.13%)	384 (13.45%)
tyrosine	6910 (29.36%)	1152 (16.67%)
total	23,536	3311 (14.07%)
Phenylalanine Interactions per Halogen		
Cl	7667 (75.36%)	806 (10.51%)
Br	1874 (18.42%)	198 (10.57%)
I	633 (6.22%)	110 (17.38%)

a The focus is on phenylalanine contacts and interactions with corresponding results reported for each halogen separately.

Since the model is supposed to assess halogen interactions onto benzene, we only focus on phenylalanine. With 10,174 (43.23%) contacts, halogen···PHE contacts are the most prevalent. To concentrate on potential halogen bonds in terms of σ-hole interactions, we applied distance and angle constraints. Furthermore, the C–X vector of the ligand must point toward the benzene plane to increase the likelihood of a σ-hole interaction. Based on these restrictions, we identified 1114 (10.95% from the initial total PHE contacts) XB interactions for subsequent analysis. In a matched molecular pair approach, ligands were replaced with a model system of the corresponding halobenzene. The halogen atom and the C–X vector are matched exactly onto the original ligand. Additionally, the plane of the benzene ring is optimally aligned onto the ligand’s aromatic ring system. The benzene ring of phenylalanine side chain residue was capped at the C_γ_–C_β_-bond and replaced by a protonated and optimized benzene system by aligning the two ring systems. It should be noted that the halobenzene model system can be much less tuned than original ligand systems where thewere calculated. Two examples were excluded, resulting scaffold or functional groups affect the σ-hole strength. The final data set consists of 1114 unique interaction geometries with 806 chlorine, 198 bromine, and 110 iodine interactions. Single-point calculations on an MP2/TZVPP level of theory were conducted using TURBOMOLE.

### Evaluation of the Model Using the PDB Set

Similar to the previous data set, adduct formation energies of the PDB set in a final set of 1112 complexes. These examples originate from the crystal structures 4HSX and 7EPZ, containing two geometries with very low distances facilitating severe clashes, and in turn, leading to repulsive energies. Geometric features were extracted from the geometries and fed into the model to assess its applicability to real-world biological examples, rather than relying solely on artificially derived data. The individual data points and energies are provided in spreadsheet format in the Supporting Information. As shown in Figure 5a, the distribution of energy differences (ΔΔ*E*) between calculated and predicted interaction energies remains centered around zero for all halogens (Cl, Br, I), with most differences falling within ±1 kJ/mol (82.22%). A maximum of ΔΔ*E* = +20.42 kJ/mol and a minimum of ΔΔ*E* = −6.49 kJ/mol was observed. The model achieves good predictive accuracy on the PDB examples, with an *R* ^2^ = 0.805 and an RMSE = 1.57 kJ/mol (Figure 5b). Despite the overall good accuracy, a few outliers (labeled A–D) still show notable prediction errors. These structures correspond to interaction geometries that lie statistically at the edge of, or outside the training feature distribution, as indicated by increased MDs. Detailed information on the four examples is given in Table 3. Figure 6 illustrates the detailed geometries of these four outlier examples (A–D) identified in the PDB data set analysis (Figure 5b). These structures show substantial deviations between predicted and calculated interaction energies. Example A displays a close contact with *d*_Br···π‑plane_ = 1.68 Å and a relatively large angle of α_C–Br···⊥(π‑plane)_ = 43.2°. The halogen, and particularly the C–X bond vector, is oriented away from the aromatic plane. This orientation places the σ-hole outside the optimal interaction direction. However, this geometry exhibits features of other interaction types, resulting in a mixed interaction profile that combines aspects of π···π stacking and lateral C–H···X contacts onto the negative belt of the halogen. The overall interaction remains attractive, but its nature differs from the purely σ-hole-driven contacts represented in the training data. Therefore, this configuration lies outside the range of training data, leading to a notable underestimation by the model. In contrast, examples B, C, and D are characterized by short halogen−π distances (*d*_Cl···π‑plane_ = 2.15 Å, 1.61 Å, and 1.25 Å, respectively) and moderate angles (α_C–Br···⊥(π‑plane)_ = 44.6°, 40.1°, and 38.3°), yet the model overestimates their interaction strength. These discrepancies likely arise from the fact that these geometries, although derived from biological examples, are underrepresented or absent in the training distribution, limiting the model’s ability to assign accurate energies. Importantly, these interaction motifs fall outside the intended scope of σ-hole interactions. In these cases, the halogen atom is positioned outside the π-system plane, or the halobenzene adopts a geometry with unrealistically short contact distances to the benzene molecule, leading to steric repulsion as confirmed by positive Δ*E*_calc_ values. Additionally, the C–X bond vector typically points away from the π-system, preventing the spatial arrangement required for a directional σ-hole interaction. The origin of these apparent steric clashes in examples B (2YLP) and C (2Q6N) is further examined in the Supporting Information, where we provide a more detailed structural analysis (Figures S2 and S3). Thus, while such geometries may appear in experimental structures, they do not constitute σ-hole interactions and are neither expected nor intended to be well captured or prioritized by the model. In addition, although the model was trained exclusively on halobenzenes, some PDB examples contain halogens bound to heteroaromatic five-membered rings. To evaluate transferability, we recalculated the interaction features using the corresponding five-ring geometries and compared the predicted energies to those obtained with the initial six-membered ring features of the halobenzene. On average, the difference between both predictions was 0.3 kJ/mol with a standard deviation of 0.44 kJ/mol, demonstrating that the model generalizes well to heteroaromatic π-systems and that the feature representation is not biased toward a particular ring type.

**Figure 5:**
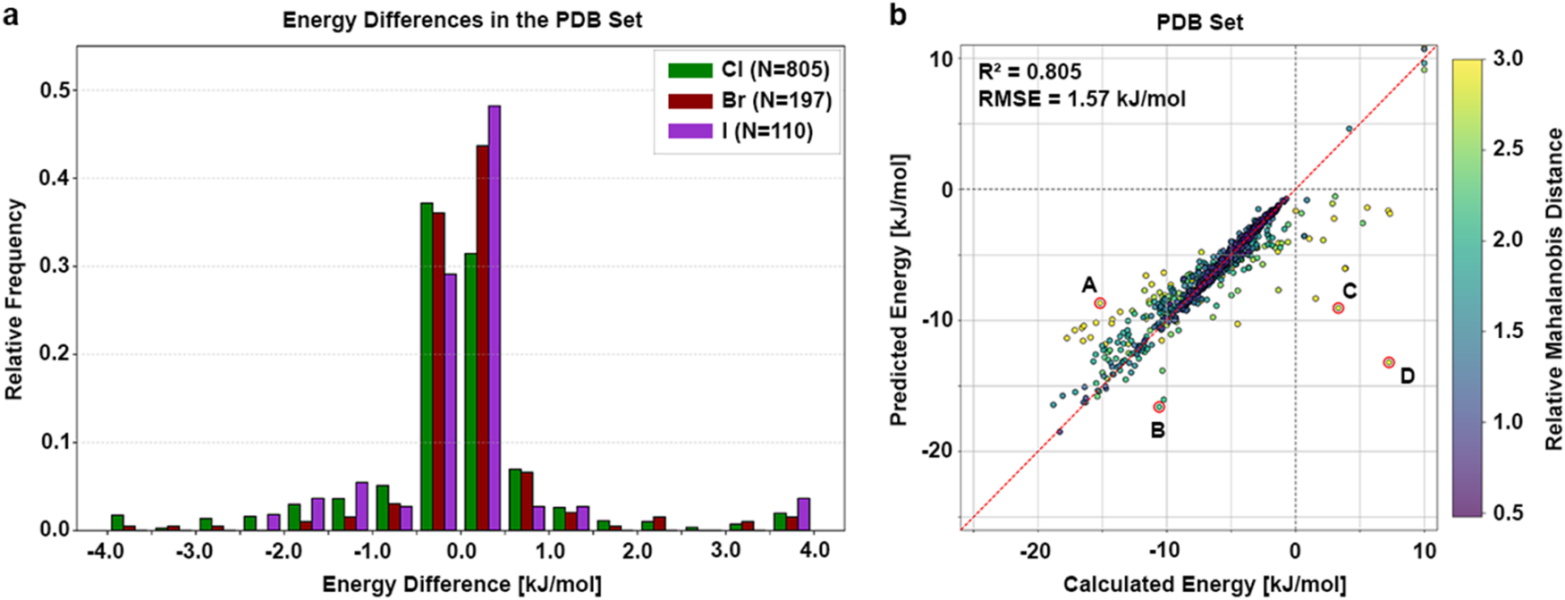
Model performance on the PDB set. (a) The histogram shows the relative frequencies of energy differences between calculated and predicted energy (total of 1112 data points) in bins of 0.5 kJ/mol from −4.0 to 4.0 kJ/mol for chlorine (green, *N* = 805), bromine (dark red, *N* = 197), and iodine (purple, *N* = 110) separately. Energy differences are calculated as ΔΔ*E* = Δ*E*_calc_ – Δ*E*_pred_. Larger values are clipped to the respective limitation for better visibility. (b) Calculated energy is plotted against the predicted energy. The model achieved an *R* ^2^ = 0.805 with an RMSE = 1.57 kJ/mol. The red, dashed line indicates the perfect correlation between calculated and predicted values, while the gray dashed lines indicate the transition from negative to positive energies. Each data point is colored according to its relative MD with respect to the given color scale. Data points outlined with a red circle and labeled with (A–D) are discussed in detail in Figure 6.

**Figure 6:**
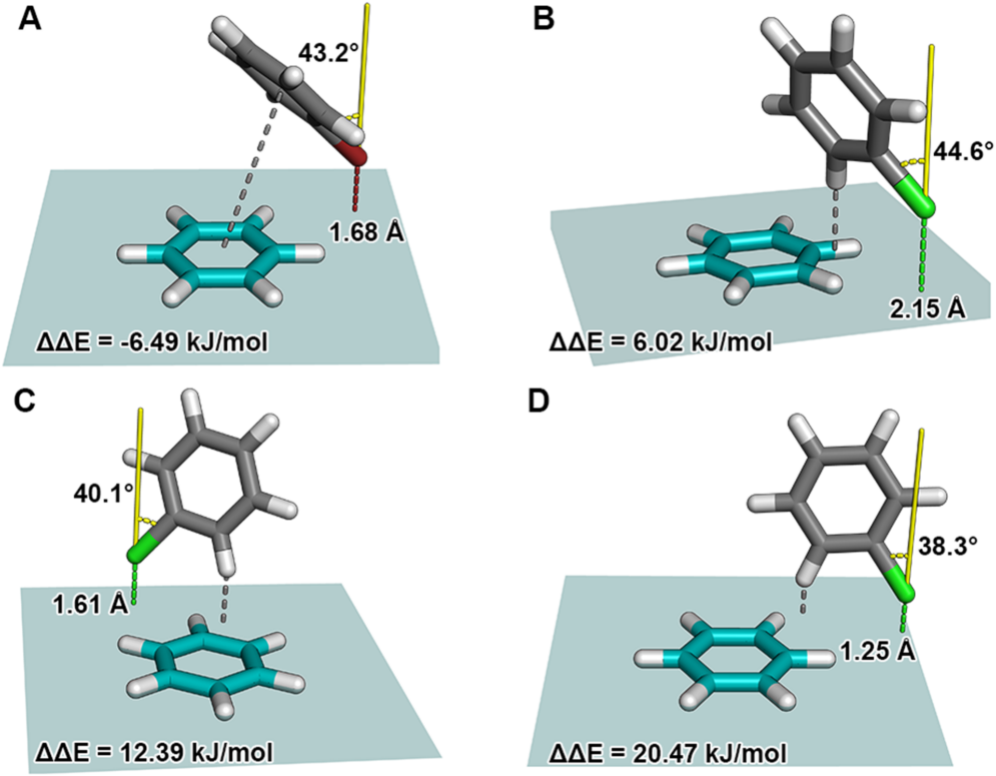
Interaction geometries corresponding to selected outliers of the PDB set identified in the scatter plot shown in Figure 5. These structures represent data points with unusually high deviations between calculated and predicted adduct formation energies. Each geometry (A–D) illustrates the spatial arrangement of the halobenzene (gray) relative to the π-system of benzene (teal), including the halogen distance *d*_X···π‑plane_ (dashed line, colored according to the halogen) to the π-system plane in Å, the torsion angle α_C–X···⊥(π‑plane)_ (colored yellow) in degrees between the C–X vector and the normal of the π-system plane, as well as the energy difference ΔΔ*E*. The gray dashed line highlights the type of interaction that may contribute to the observed prediction error. For better visibility, the teal-colored plane illustrates the benzene plane and the dimensions of the models’ training grid. (A) Bromobenzene interaction with *d*_Br···π‑plane_ = 1.68 Å and α_C–Br···⊥(π‑plane)_ = 43.2° The gray dashed line indicates a π···π interaction. (B) Chlorobenzene interaction with *d*_Cl···π‑plane_ = 2.15 Å and α_C–Cl···⊥(π‑plane)_ = 44.7°. The gray dashed line indicates a C–H···π contact. (C) Chlorobenzene interaction with *d*_Cl···π‑plane_ = 1.61 Å and α_C–Cl···⊥(π‑plane)_ = 40.1°. The gray dashed line indicates a C–H···π contact. (D) Chlorobenzene interaction with *d*_Cl···π‑plane_ = 1.25 Å and α_C–Cl···⊥(π‑plane)_ = 38.3°. The gray dashed line indicates a C–H···π contact.

**Table 3:** Overview of Energy Values and Geometric Parameters for Outlier Structures (A–D) (from the PDB Set) Highlighted in Figure 5 and Depicted in Figure 6 ^a^

	halogen	PDB ID	Δ*E*_calc_ [kJ/mol]	Δ*E*_pred_ [kJ/mol]	ΔΔ*E* [kJ/mol]	*d*_X···π‑plane_ [Å]	α_C–X···⊥(π‑plane)_ [deg]
A	Br	5RTQ	–15.17	–8.68	–6.49	1.68	43.2
B	Cl	5CU3	–10.58	–16.6	6.02	2.15	44.6
C	Cl	2YLP	3.33	–9.06	12.39	1.61	40.1
D	Cl	2Q6N	7.26	–13.2	20.47	1.25	38.3

a The halogen symbolizes the interacting halobenzene. The PDB ID indicates the crystal structure the interaction geometry was extracted from. Values of calculated and predicted energies, as well as the difference between both are given in kJ/mol. Distance values *d*_X···π‑plane_ between the halogen and the π-plane are given in Å. Angle values between the C–X vector and the normal to the π-plane are given in degree [deg].

For practical applications such as docking algorithms or virtual screening, additional empirical terms for π···π or C–H···π interactions would be required to properly capture these motifs. Addressing such effects lies beyond the present scope of this study. However, repulsive geometric arrangements are typically already handled by standard distance-dependent terms in scoring functions, while attractive motifs such as π···π or C–H···π interactions should or could be incorporated through dedicated scoring functions or extensions of existing ones.

### Conclusion and Outlook

In this proof-of-concept study, we have employed neural networks (NNs) to predict the interaction strength of halogen···π interactions between halobenzenes (chlorobenzene, bromobenzene, iodobenzene) and a simple benzene model. Nearly 1.4 million single-point calculations were conducted and adduct formation energies calculated. NN models were trained on geometric features extracted from the interaction geometries. Extensive hyperparameter tuning was used to find the most suitable model configuration. Model validation was carried out using stratified 5-fold cross-validation and further tested on independent data sets. The model demonstrated excellent predictive performance on the cross-validation set, achieving an *R* ^2^ = 0.998 and a very low RMSE = 0.16 kJ/mol, a level of accuracy that likely approaches the intrinsic limits of computability of interaction energies. These results reflect the model’s ability to interpolate accurately within the feature space of the training data. When applied to a separate random geometry test set, the model still performed strongly with *R* ^2^ = 0.86 and RMSE = 1.33 kJ/mol, while similar results were obtained for the PDB-derived data set with *R* ^2^ = 0.81 and RMSE = 1.57 kJ/mol. Most predictions fall within a narrow error range, demonstrating the model’s ability to generalize across a wide variety of geometries. Analysis of outliers revealed that large prediction errors occur primarily when input features lie far beyond the training distribution. In such cases, the model either fails to recognize favorable π···π interaction motifs or misinterprets repulsive C–H···π contacts, which were not explicitly covered during training. Nonetheless, these geometries are not representative of the directional σ-hole interactions the model was designed to capture and can therefore be neglected or are covered by other terms. Nevertheless, incorporating the repulsive part of σ-hole interactions in future developments would improve the robustness of the model and extend its applicability beyond the attractive interaction space. Overall, this study demonstrates that NNs trained on well-defined and well-chosen geometric features can reliably predict halogen···π interaction energies within their defined purpose. The approach offers a fast and accurate alternative to quantum chemical calculations. By leveraging the methodological “double jump” from CCSD­(T) → MP2 → NNs, the model retains accuracy close to CCSD­(T) benchmarks while achieving a runtime speed-up of up to 8 orders of magnitude compared to MP2 calculations. However, this study represents an initial step involving only a single aromatic residue model. As a next step, we aim to extend this approach to the remaining aromatic amino acid side chain residues of histidine, tyrosine, and tryptophan, for which data generation and model development are already underway. By constructing exclusive NN models for each of these residues, we seek to capture the diversity of biologically relevant halogen···π interactions. Ultimately, these models will be integrated into the molecular docking framework PLANTS, enabling improved recognition and scoring of halogen bonding interactions in protein–ligand complexes. Additionally, we aim to gradually integrate the models within the Galaxy Webserver^58^ as standalone scoring function modules. Uploaded molecular structures will be systematically analyzed to detect halogen···π interactions, and, if present, the interaction strengths will be quantitatively evaluated.

## Materials and Methods

### Structure Optimization

Geometry optimizations of the individual ligand model systems (iodobenzene, bromobenzene, and chlorobenzene) and the amino acid model system (benzene) were done at the MP2 ^59,60^ -level of theory using TURBOMOLE 7.7.1 ^61^ with a triple-ζ basis set (def2-TZVPP^62^) on the JUSTUS2–bwHPC Cluster.^63^ Calculations were done in combination with the resolution of identity (RI) technique and the frozen core approximation. Frozen core orbitals were defined using default settings, where orbitals with energies below −3.0 au are considered core orbitals. SCF convergence criterion was increased to 10^–8^ hartree. Relativistic effects for iodine were considered by an effective core potential (ECP).^64–72^

### Generation of Interaction Geometries

Interaction geometries of chloro-, bromo-, and iodobenzene in complex with benzene were generated. Halobenzenes were placed on a regular grid using *X*- and *Z*-translations (Figure 7a) for eight different distances (Figure 7b). Following previous approaches, an optimal σ-hole angle of α_C‑X···π‑plane_ = 180° was initially used. To capture rotational features, the halobenzene itself was rotated around the *y*-axis. Furthermore, the σ-hole angle was altered to deviations from −40° to 40° in steps of 10° in the *x*-, and *z*-directions (Figure 7d). Two additional rotation axes were incorporated, lying in 45° to the *x*-, or *z*-axis (Figure 7e). Due to the symmetric nature of benzene, only one quadrant of the grid was considered. With this systematic procedure, 508,032 interaction geometries per halobenzene were generated. A total of 1,397,247 single point calculations were conducted on an MP2/TZVPP level of theory. Adduct formation energies were calculated as

1 $$\Delta E_{\mathrm{calc}}=\left(E_{\mathrm{complex}}-\left(E_{\mathrm{halobenzene}}+E_{\mathrm{benzene}}\right)\right)$$

and reported as kJ/mol.

**Figure 7:**
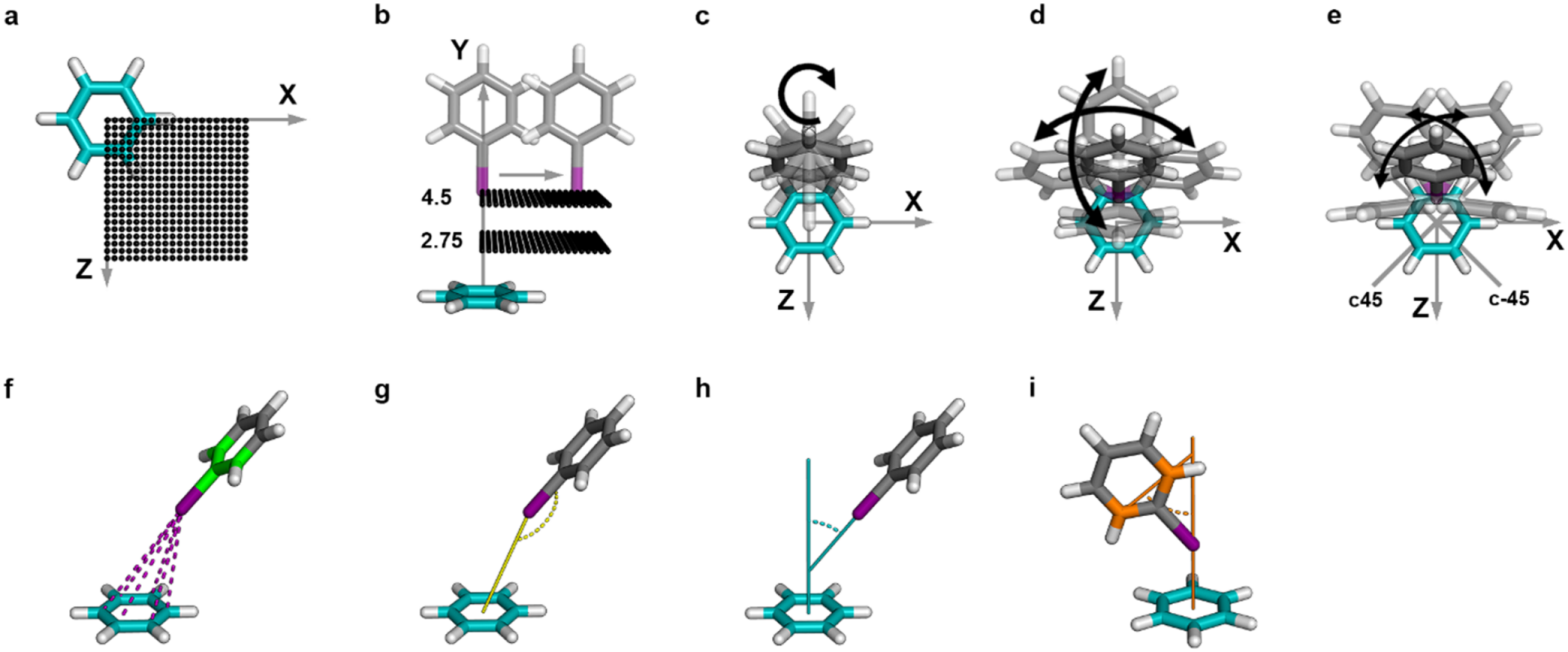
Overview of the interaction geometry generation and the feature extraction. (a) Grid points on the *XZ*-plane were generated with dimensions *X*_translation_ = [0.0 Å to 5.0 Å], *Z*_translation_ = [0.0 Å to 5.0 Å] in steps of 0.25 Å. (b) Grid points were generated for eight different distances, *d*_X···π‑plane_ = [2.75 Å to 4.5 Å] in steps of 0.25 Å, between the halogen atom (Cl, Br, or I) and the benzene plane. (c) Rotations of the halobenzene around the *y*-axis *y*_rot_ = [0° (initially), 45°, 90°, 135°]. (d) Deviations from the optimal σ-hole angle α_C–X···π‑plane_ = 180° from −40° to 40° in steps of 10° achieved by rotating around the *x*- and *z*-axis. (e) Custom-generated rotational axis (c45 and c-45), lying in 45° to the *x*-, and *z*-axis. Rotations around these axes similar to (d). (f) Pairwise distances from the halogen atom and the green colored carbons of the halobenzene to all carbon atoms of the benzene are extracted as features. (g) Angle feature of the halogen atom, its neighboring carbon and the center of mass of the benzene, α_C–X···CoM(benzene)_ . (h) Angle feature of the C–X vector and the normal of the benzene plane α_C–X···⊥(π‑plane)_. (i) Angle feature of the vector between the orange-colored carbons of the halobenzene and the normal of the benzene plane.

### Feature Extraction and NN Model Training

All data preprocessing, feature extraction, and learning approaches were built in Python using custom scripts with the *PyTorch* ^73^ and *scikit-learn* package, two open-source Python libraries for machine learning. NN models were trained on geometric features extracted from the interaction geometries. The training process was performed on the BinAC–bwHPC Cluster.^74^ The feature vector **v⃗** *=* (*d*_1_, *d*_2_, ..., *d*_*n*_, *a*_1_, *a*_2_, *a*_3_) consists of 30 features. Table S1 shows details of all features. The adduct formation energy Δ*E* of each geometry serves as the target value. Distance features are calculated as pairwise distances between selected atoms of the halobenzene (halogen atom, its neighboring carbon atom, and from this carbon two adjacent carbon atoms) and the benzene system (Figure 7f). The features are sorted according to the distances between halogen and benzene carbon atoms in ascending order, such that the nearest distance is defined as *d*_X···C_(min) = ***v⃗*** (d_1_), the second-shortest distance as ***v⃗*** (d_2_), and so forth. The distances between the benzene carbons and the halobenzene carbons follow the same ordering as the halogen–carbon distances. Three angle features are calculated between vectors of the halobenzene and the benzene plane. One angle is calculated between the halogen atom, its neighboring carbon atom, and the center of mass of the benzene (Figure 7g). A second angle is calculated between the C–X vector and the normal of the benzene plane (Figure 7h). And a third is calculated between two carbon atoms of the halobenzene and the normal of the benzene plane (Figure 7i). Before training, all features were individually normalized using a min-max scaler (scikit-learn MinMaxScaler). The data set was split into training (80% of the data) and test set (20%) in a stratified leave-one-out 5-fold cross validation approach to ensure model consistency (Figure 8). Stratification was applied using (1) the halogen, and (2) distances between halogen and π-plane to ensure features were equally distributed and divided across the corresponding sets. Subsequently, the training set was further split into model training (80%) and model validation (20%) using the same stratification strategy. Extensive hyperparameter tuning was performed on the training data set within a supervised learning approach. This process involved systematic variation of activation functions (Sigmoid, Tanh, and Leaky ReLU, as provided by *PyTorch*), the number of hidden layers (ranging from two to six), and the sizes of hidden layers using combinations from the set [256, 128, 64, 32, 16, 8] in a fully connected feed-forward network. Additional parameters included initial learning rates (10^–1^ to 10^–5^), batch sizes (32, 64, 128, 256), and the number of training epochs (10 to 1000). Model training utilized the Adam optimizer (*PyTorch* built-in), an elastic-net-weighted-MSE loss function to address data imbalance, and early stopping criteria to prevent overfitting. The combination yielding the best performance on the validation set was selected for final training and evaluation. The mean absolute error (MAE), the root-mean-square error (RMSE), and the coefficient of determination (*R* ^2^) were employed to evaluate the predictive performance of the models. An *R* ^2^ value approaching 1.00, alongside low MAE and RMSE values, indicates that a model achieves high predictive accuracy. Energy differences between calculated and predicted adduct formation energies are reported as

2 $$\Delta \Delta E=\Delta E_{\mathrm{calculated}}-\Delta E_{\mathrm{predicted}}$$

in kJ/mol. The training started with a fixed learning rate and was adapted (value was halved) up to a minimum of 0.0001 if there had not been a loss improvement for 10 epochs. The final model consists of three internal hidden layers (of size: 64, 32, 16) with Leaky Rectified Linear Unit (Leaky ReLU) as activation functions, an initial learning rate of 0.01, and was trained on a batch size of 128.

**Figure 8:**
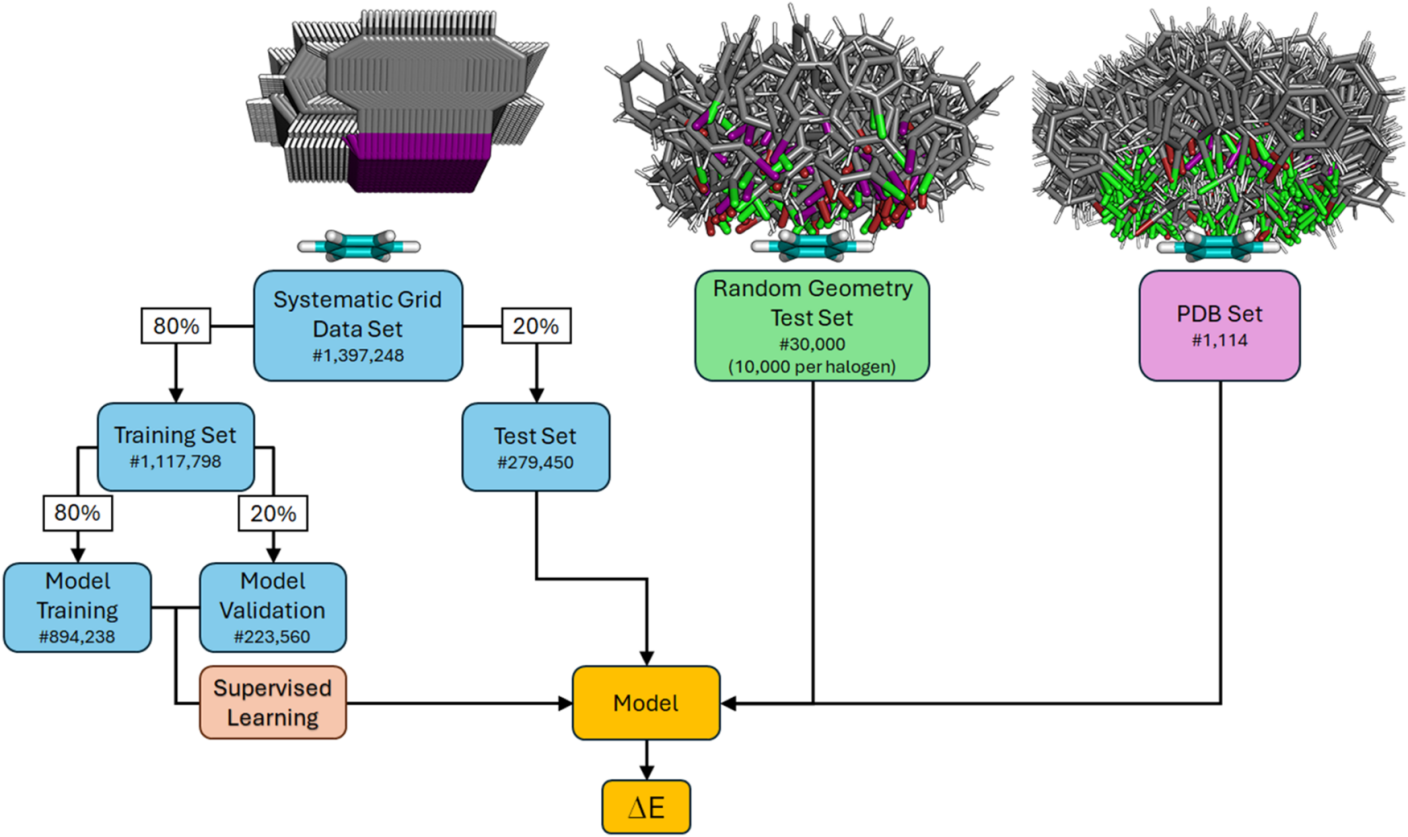
Overview of the different data sets. The systematic grid data set (1,397,248 geometries) is split into training (80% of the whole data set, 1,117,798 geometries) and test set (20%, 279,450 geometries). The training set is further split into model training (again 80%, 894,236 geometries) and model validation set (20%, 223,560 geometries). The two model data sets are fed into the model in a supervised training approach. The random geometry test set (30,000 geometries) is used to evaluate the models’ generalized performance on unseen data. The PDB set (1114 geometries) is used to represent and evaluate biological examples. The respective geometries shown are only a small excerpt of the full data sets.

### Generation of a Random Geometry Test Set

Similar to the generation of the systematic data set used for the training, we generated a smaller subset of 30,000 interaction geometries (10,000 per halobenzene) with random geometric features to test the model’s generalized performance on unseen data. Therefore, parameters of *X*,*Z*_translation_ = [−5.0 Å to 5.0 Å], *Y*_translation_ = [1.5 Å to 5.0 Å], *y*_rot_ = [0° to 360°], α_C–X···⊥(π‑plane)_ = [0° to 60°] were randomly chosen and applied to a halobenzene. To ensure uniqueness of the generated geometries, newly chosen parameters were compared to previous ones, and only applied if found to be distinct.

Such a more inclusive definition of broad angular and distance limits was employed to capture a diverse range of halogen···π interaction geometries, including those near the characteristic boundaries of σ-hole interactions. Our aim is to evaluate the model’s generalizability and highlight its potential limitations.

### PDB Scan of Phenylalanine Acceptors

A PDB (as of July 2025:239,149) scan was conducted using a custom Python/PyMOL^75^ script. Alternative conformations, metal ions and hydrogen atoms were removed from the structure. PDB structures containing halogenated ligands (chlorine, bromine, iodine) with the halogen atom connected to an aromatic ring system and a ligand size of six or more heavy atoms were considered for further analysis. Aromatic amino acid side chain residues of phenylalanine within 5 Å of the halogen atom were retained as XB acceptors. As an additional constraint, the C–X vector must point toward the benzene plane (α_C–X···⊥(π‑plane)_ < 50°) increasing the likelihood of a potential halogen bond. As lower distance limit we chose a minimal distance of *d*_X···AA_= 1 Å. The side chain residue was replaced by a benzene molecule. For simplicity, we replaced each ligand with the corresponding halobenzene (chloro-, bromo, or iodobenzene) by matching the halogen atom, the C–X bond vector and the plane of the (hetero)­aromatic system in a matched molecular pair approach. Both molecules were separately geometry optimized on an MP2/TZVPP level of theory as previously described. A total of 1114 interaction geometries of the halobenzene in complex with the addressed benzene were extracted to carry out a single point calculation on an MP2/TZVPP level of theory. They again represent a more inclusive set of diverse geometries, even at the border of typical halogen bonds. Adduct formation energies were calculated using the supermolecular approach.

### Model Evaluation and Outlier Detection

To evaluate the model’s ability to predict adduct formation energies of a given interaction geometry we use the three data sets (20% test set, random geometry set, and PDB set). Ideally, the data sets consist of “unseen” data for the model to test its’ generalizability. Similar to the training process, we examined the results of the prediction using the root-mean-square error (RMSE) and the coefficient of determination (*R* ^2^). Given that interaction geometries in the random geometry set and the PDB set can lie outside the feature space of the training set, the Mahalanobis Distance^76^ (MD), a statistical measure of dissimilarity between each test point and the center of the multivariate distribution of training features, was employed to assess the degree to which a given data point deviates from the input space. It is defined as

3 $$D_{M}\left(x\right)=\sqrt{{\left(x-\mu \right)}^{T}\Sigma^{-1}\left(x-\mu \right)}$$

where *x* is the feature vector of the data point, μ is the mean vector of the training data, and ∑ is the covariance matrix of the training data. High distances indicate that a geometry lies in a statistically rare region of input space relative to the training set, thus identifying it as a potential extrapolation point or outlier. Relative MDs were computed for all geometries in the test sets, and a threshold for outlier classification was set based on the 95th percentile of the distribution of distances within the training set.

## Supplementary Material





## Data Availability

PyMOL is an open-source software maintained and distributed by Schrödinger. There is an open-source version of PyMOL available at: https://github.com/schrodinger/pymol-open-source. Python and all of its’ packages is an open-source programming language available and downloadable from https://www.python.org/. PyTorch is an open-source machine learning library for Python: https://pytorch.org/. TURBOMOLE is a purchasable software maintained and distributed by the *TURBOMOLE GmbH*. Demo versions are available at https://www.turbomole.org/. The licensed software was provided to us by the bwHPC Cluster JUSTUS2.

## Notes

Graphical depiction of the distance-based features; Detailed description of the features used for training; Structural analysis of PDB examples 2YLP, 26QN (PDF)
Detailed tables of the individual data points of the random geometry data set and the PDB data set with corresponding adduct formation energies for calculated and predicted energies (XLSX)

## Abbreviations

XB: halogen bond
X: halogen
QM: quantum mechanical
MP2: Møller–Plesset perturbation method order 2
TZVPP: valence triple-ζ with two sets of polarization functions
cc-pVTZ: correlation-consistent polarized valence triple-ζ function
SCF: self-consistent field
RI: resolution of identity
RMSE: root mean square error
ECP: effective core potential
NN: neural network
R^2^: coefficient of determination
MD: Mahalanobis Distance
PDB: Protein Data Bank
PHE: phenylalanine
MAE: mean absolute error
MSE: mean squared error
CCSD­(T): coupled cluster with single, double, and perturbative triple excitations
