# Supplementary material for: A QM-AI Approach for the Acceleration of Accurate Assessments of Halogen‑π Interactions by Training Neural Networks
Source: J Chem Inf Model. 2025 Nov 25;65(24):13132–44. doi: 10.1021/acs.jcim.5c02136 (PMC12728935; doi:10.1021/acs.jcim.5c02136)
Supplement: Supplementary file 1 [file ci5c02136_si_001.pdf]

# Supporting Information

# A QM-AI Approach for the Acceleration of Accurate Assessments of Halogen-π Interactions by Training Neural Networks

*Marc U. Engelhardt[1], Finn Mier[1], Markus O. Zimmermann[1,2], Frank M. Boeckler[1,2,*]*

[1] Laboratory for Molecular Design & Pharmaceutical Biophysics, Institute of Pharmaceutical Sciences, Department of Pharmacy and Biochemistry, Eberhard Karls Universität Tübingen, 72076 Tübingen, Germany.

[2] Interfaculty Institute for Biomedical Informatics (IBMI), Eberhard Karls Universität Tübingen, 72076 Tübingen, Germany.

Corresponding Author

*Frank M. Boeckler: frank.boeckler@uni-tuebingen.de

**This file contains the following information:**

**Supplementary Figures:**

- Supplementary Figure S1: Depiction of distance-based features of halobenzene and benzene
- Supplementary Figure S2: Binding site section of the crystal structure 2YLP
- Supplementary Figure S3: Binding site section of the crystal structure 26QN

**Supplementary Tables:**

- Supplementary Table S1: Overview of the features derived from interaction geometries

**Supplementary Analysis:**

- Structural Analysis of the PDB structures 2YLP and 26QN

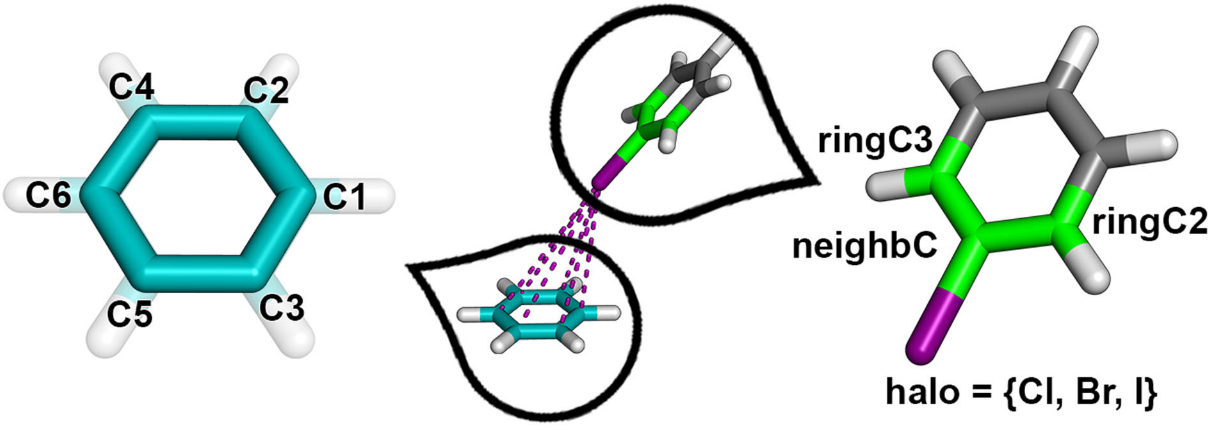


**Figure S1.** Depiction of distance-based features and the corresponding atom-naming conventions for benzene (left) and halobenzene (right, shown here as iodobenzene). The central illustration highlights the pairwise distances between the halogen and each carbon atom of the benzene. In the detailed views, benzene carbon atoms are labeled according to their ascending distance from the halogen (C1 = nearest, C2 = second nearest, and so forth). In halobenzene, the carbon directly bonded to the halogen is labeled neighbC, while ringC2 and ringC3 denote the nearest carbons in the aromatic ring to that neighbor.

**Table S1:** Overview of the features derived from the interaction geometries. Feature names follow the atom-naming scheme shown in Figure S1. Distances are reported in Å, and angles in degrees. The final three features are Boolean values indicating the presence of the respective halogen.

| Feature Name | Description |
|---|---|
| min_C1_halo | Distance halogen to the nearest benzene carbon C1 |
| min_C2_halo | Distance halogen to the 2nd nearest benzene carbon C2 |
| min_C3_halo | Distance halogen to the 3rd nearest benzene carbon C3 |
| min_C4_halo | Distance halogen to the 4th nearest benzene carbon C4 |
| min_C5_halo | Distance halogen to the 5th nearest benzene carbon C5 |
| min_C6_halo | Distance halogen to the furthest benzene carbon C6 |
| min_C1_neighbC | Distance benzene-carbon C1 (according to halogen distance order) and the neighboring carbon (neighbC) of the halogen |
| min_C1_ringC2 | Distance benzene-carbon C1(according to halogen distance order) and a ring carbon C2 (ringC2) of the halobenzene |
| min_C1_ringC3 | Distance benzene-carbon C1 (according to halogen distance order) and a ring carbon C3 (ringC3) of the halobenzene |
| min_C2_neighbC | Distance C2 and the neighbC |
| min_C2_ringC2 | Distance C2 and the ringC2 |
| min_C2_ringC3 | Distance C2 and the ringC3 |
| min_C3_neighbC | Distance C3 and the neighbC |
| min_C3_ringC2 | Distance C3 and the ringC2 |
| min_C3_ringC3 | Distance C3 and the ringC3 |
| min_C4_neighbC | Distance C4 and the neighbC |
| min_C4_ringC2 | Distance C4 and the ringC2 |
| min_C4_ringC3 | Distance C4 and the ringC3 |
| min_C5_neighbC | Distance C5 and the neighbC |
| min_C5_ringC2 | Distance C5 and the ringC2 |

| | |
|---|---|
| min_C5_ringC3 | Distance C5 and the ringC3 |
| min_C6_neighbC | Distance C6 and the neighbC |
| min_C6_ringC2 | Distance C6 and the ringC2 |
| min_C6_ringC3 | Distance C6 and the ringC3 |
| Angle1_CoM_L-X_L-neighbC | Angle between the C-X vector (of neighbC and the halogen) and the benzenes center of mass |
| Angle2_benz-plane_L-X_L-neighbC | Angle between the C-X vector and the normal of the benzene plane |
| Angle3_benz-plane_L-ringC2-C3 | Angle between the ringC2-ringC3 vector and the normale of the benzene plane |
| is_Cl | 1 if X = Cl, else 0 |
| is_Br | 1 if X = Br, else 0 |
| is_I | 1 if X = I, else 0 |

**Structural Analysis of PDB Examples**

**2YLP**

The crystal structure 2YLP depicts the androgen receptor in complex with the original halogenated ligand (056). The structure was determined at a resolution of 2.3 Å, which provides reliable information on the overall tertiary structure and side-chain orientations, though higher-resolution data would further improve atomic precision. The electron density for the ligand appears less well defined (Figure S2), which introduces some uncertainty in the placement of the molecule within the binding pocket. In particular, the modeled ligand orientation suggests a close contact with PHE826, consistent with the steric clash highlighted in the main manuscript. It is possible that this contact reflects limitations in the electron density map rather than a genuine interaction, and the structural details of this complex should therefore be interpreted with appropriate caution. Thus, the interaction geometry of example C (Figure 6 C), although directly extracted from the crystal structure 2YLP, may not reliably reflect the true binding motif the ligand can occupy.

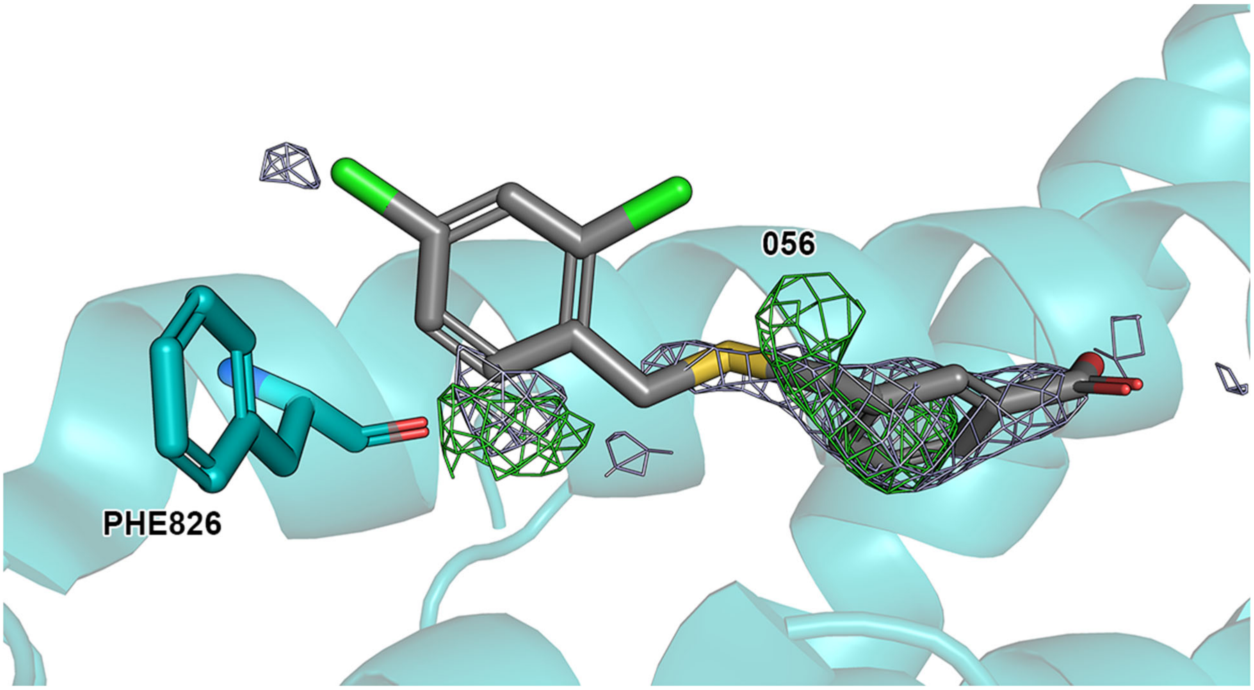


**Figure S2.** Binding site section of the crystal structure 2YLP of androgen receptor protein in complex with the original halogenated ligand 056. The electron density of the ligand is displayed as an isomesh. The green and red difference electron density ($mF_o$-$DF_c$) is contoured at ± 3 σ, while the blue $2mF_o$-$DF_c$ map is contoured at 1 σ.

**2Q6N**

The crystal structure 2Q6N presents the complex of cytochrome P450 with the small ligand 1CI, determined at a resolution of 3.2 Å. While the overall electron density fits the model well, the limited resolution combined with the inherent flexibility of such a small ligand means that its exact orientation within the binding site cannot be defined with high precision. The binding motif is dominated by the coordination of the heme group and its central iron atom, which provides strong anchoring and largely dictates the binding mode. Within this context, the apparent repulsion between PHE297 and the ligand (example D in the main manuscript) may be tolerated or compensated by the stabilizing effect of the iron coordination, and the detailed geometry of this interaction should therefore not be overinterpreted.

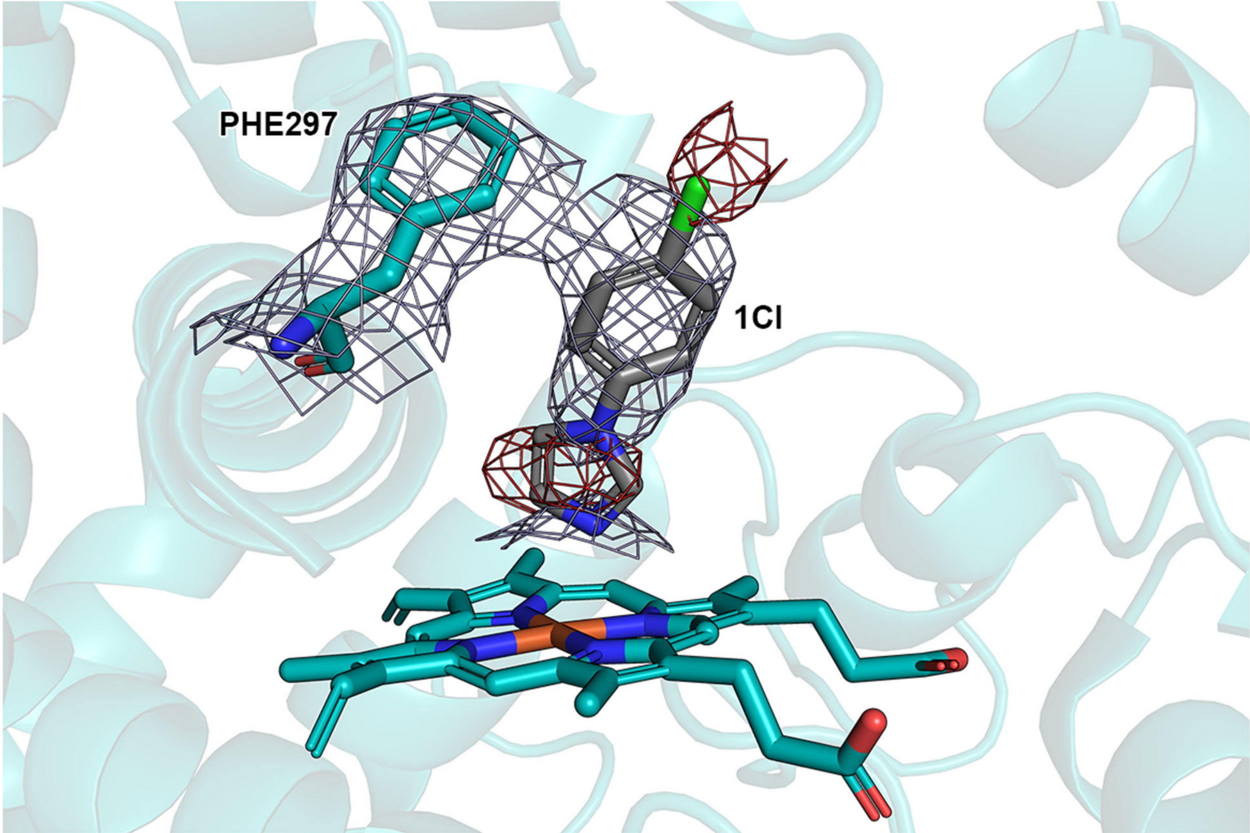


**Figure S3.** Binding site section of the crystal structure 26QN of cytochrome P450 in complex with the original halogenated ligand 1CI. The electron density of the ligand and the phenylalanine is displayed as an isomesh. The green and red difference electron density ($mF_o$-$DF_c$) is contoured at ± 3 σ, while the blue $2mF_o$-$DF_c$ map is contoured at 1 σ.
